# TREATMENT OF LATERAL EPICONDYLITIS OF THE ELBOW WITH HYALURONIC ACID INJECTIONS

**DOI:** 10.1590/1413-785220253302e289985

**Published:** 2025-10-13

**Authors:** MAURO EMILIO CONFORTO GRACITELLI, LUIZA DE CAMPOS MOREIRA DA SILVA, JORGE HENRIQUE ASSUNÇÃO, FERNANDO BRANDÃO DE ANDRADE E SILVA, ARNALDO AMADO FERREIRA, EDUARDO ANGELI MALAVOLTA

**Affiliations:** 1. Universidade de Sao Paulo, Faculdade de Medicina, Hospital das Clinicas (HCFMUSP), Sao Paulo, SP, Brazil.; 2. DASA, Hospital 9 de Julho, São Paulo, SP, Brazil.; 3. Hospital do Coracao (Hcor), São Paulo, Brazil.

**Keywords:** Tennis Elbow, Lateral Epicondylitis, Hyaluronic Acid, Viscosupplementation, Cotovelo de Tenista, Epicondilite Lateral, Ácido Hialurônico, Viscossuplementação

## Abstract

**Introduction:**

The primary objective was to evaluate the efficacy of hyaluronic acid injections for lateral epicondylitis. Secondary objectives included assessing pain and functional outcomes at various time points following the injection.

**Methods:**

This prospective cohort study included patients who received two hyaluronic acid injections one week apart after prior conservative treatment. Assessments were conducted at two and six weeks, and at three, six, and 12 months post-injection. Outcome measures were pain scores (Visual Analogue Scale), functional assessment (Single Assessment Numeric Evaluation), and injection site complications.

**Results:**

A total of 46 patients (52 elbows) were included. Significant improvements were observed in pain scores measured by VAS at rest, from 4.5±2.8 initially to 2.7±2.8 at 3 months and 2.3±3.0 at 12 months (p<0.001). Similar improvements were seen in VAS scores during maximum hand grip strength, from 5.8±3.1 initially to 2.8±3.4 at 12 months (p<0.001). SANE score also improved significantly. No complications were reported.

**Conclusions:**

Treatment of lateral epicondylitis with hyaluronic acid showed statistically significant improvement in VAS scores at rest and during maximum hand grip strength, as well as in SANE scores, with no reported complications. Level of Evidence IV; Case Series.

## RESUMO

**Introdução:**

O objetivo primário foi avaliar a eficácia das infiltrações com ácido hialurônico para epicondilite lateral. Os objetivos secundários incluíram avaliar a dor e os resultados funcionais em vários momentos após a infiltração.

**Métodos:**

Este estudo de coorte prospectivo incluiu pacientes que receberam duas injeções de ácido hialurônico com uma semana de intervalo após tratamento conservador prévio. As avaliações foram conduzidas em duas e seis semanas, e em três, seis e 12 meses após a infiltração. As medidas de desfecho foram escores de dor (Visual Analogue Scale), avaliação funcional (Single Assessment Numeric Evaluation) e complicações no local da infiltração.

**Resultados:**

Um total de 46 pacientes (52 cotovelos) foram incluídos. Melhoras significativas foram observadas nos escores de dor medidos pela EVA em repouso, de 4,5 ± 2,8 inicialmente para 2,7 ± 2,8 em 3 meses e 2,3 ± 3,0 em 12 meses (p < 0,001). Melhoras semelhantes foram observadas nos escores VAS durante a força máxima de preensão manual, de 5,8 ± 3,1 inicialmente para 2,8 ± 3,4 em 12 meses (p < 0,001). O escore SANE também melhorou significativamente. Não foram relatadas complicações.

**Conclusões:**

O tratamento da epicondilite lateral com ácido hialurônico apresentou melhora estatisticamente significativa nos escores VAS em repouso e durante a força máxima de preensão manual, bem como nos escores SANE, sem complicações relatadas. Nível de Evidência IV; Série de Casos.

## INTRODUCTION

Lateral epicondylitis is a common cause of elbow pain, affecting approximately 1 to 3% of the population annually.^1^ Studies show that about 80% of cases experience symptomatic improvement within one year.^2,3^ Conservative treatment is the cornerstone approach for managing lateral epicondylitis.^4^ However, it can often be protracted and restrictive for both daily activities and sports participation.^5^

Despite the widespread adoption of conservative measures such as rest, physiotherapy and nonsteroidal anti-inflammatory drugs, a significant proportion of patients experience persistent symptoms and functional limitations. Between 4 to 11% of patients develop chronic symptoms requiring surgical intervention.^2,6-8^ This underscores the search for adjunctive therapeutic modalities to enhance conservative treatment outcomes.

Several types of injections have been explored in the management of lateral epicondylitis, including corticosteroid injections, hialuronic acid, platelet-rich plasma (PRP), and autologous blood injections. A meta-analysis by Krogh et al.^9^ compared the efficacy of corticosteroid injections, PRP, and placebo for the treatment of lateral epicondylitis. The analysis revealed that corticosteroid injections provided short-term pain relief but were associated with a higher risk of symptom recurrence compared to PRP and placebo. PRP injections demonstrated superior long-term outcomes in terms of pain reduction and functional improvement compared to corticosteroid injections, albeit with a higher cost and increased risk of complications, such as local hematoma or infection.

In light of the limitations and potential adverse effects associated with corticosteroid injections and the higher cost of PRP, alternative approaches such as hyaluronic acid injections (HA) have gained attention. Petrella et al.^10^ conducted a randomized trial evaluating the efficacy of HA in comparison to corticosteroid injections for lateral epicondylitis. The results demonstrated comparable short-term pain relief between HA and corticosteroid injections, with HA showing a lower recurrence rate and fewer adverse effects. Given these findings, our approach leans towards considering HA as a viable alternative to corticosteroid injections, particularly in patients who are refractory to conservative management or have concerns regarding the potential adverse effects associated with corticosteroid use.

### Objectives

Primary objective: to analyze the functional results, according to the visual analogue scale (VAS) at rest of patients undergoing treatment with hyaluronic acid injections after 3 months.

Secondary objectives: evaluate the VAS scale at rest at 2 and 6 weeks, after 6 and 12 months, the VAS scale during maximum hand grip strength and the Single Assessment Numeric Evaluation (SANE) scale at 2 and 6 weeks, 3, 6 and 12 months.

## METHODS

### Study design

Patients with lateral epicondylitis were included in a prospective case series, after signing the informed consent. The patients were treated by doctors certified by the Brazilian Society of Shoulder and Elbow Surgery. The study was approved by the local ethical committee (CAAE 32016620.4.0000.0068).

### Population

Patients older than 18 years and with clinical diagnosis of lateral epicondylitis, with pain on the lateral aspect of the elbow and in the lateral epicondyle, and positive clinical Cozen and Mills tests and imaging examination (ultrasound or magnetic resonance imaging) with signs of lateral epicondylitis were included in the study. Patients should have undergone conservative treatment for at least 2 months before enrollment.

Non-inclusion criteria were previous history of fracture or dislocation, osteoarthritis or focal cartilage lesion, neurological injury, previous surgery for epicondylitis, or any surgery on the upper limb, lack of mental capacity to understand the questionnaires, and active or previous infection in the affected elbow. Exclusion criteria comprised loss to follow-up before the first assessment, at 1 month.

### Interventions

Included patients underwent two injections for lateral epicondylitis with hyaluronic acid, 1 week apart. Sportvis® hyaluronic acid was used (Biolab®) (12mg in 1.2ml of 1% sodium hyaluronate in a phosphate-buffered solution, biocompatible for periarticular infiltration in soft tissues).

Injection was performed 1 cm distal to the lateral epicondyle, at the point of greatest pain, using the technique described by Petrella et al.^10^ The usual sterile preparation was performed and a local anesthetic injection was applied with 2% lidocaine without vasoconstrictor. The hyaluronic acid injection was performed next, with a 30x7 needle and distributed in at least 2 points around the initial insertion point. A second injection was administered in the same manner after 2 weeks.

In the following 2 weeks, the patient was instructed to use elbow, wrist and hand mobilization, but avoiding exertion with the affected limb. No type of immobilization was recommended.

The medication protocol was the same for all patients and consisted of Dypirone 1g 6/6h and Diclofenac sodium 8/8h for 3 days after the first and second infections. Patients were instructed to perform home stretches for the forearm extensor muscles for 2 weeks after the first injection.

### Outcomes and Variables

The outcomes were evaluated before treatment and after inclusion in the study, after 2 and 6 weeks, 3, 6 and 12 months. The scores comprised the visual analogue scale (VAS) at rest, the VAS scale during maximum hand grip strength, and the Single Assessment Numeric Evaluation (SANE).

Additionally, assessments included an evaluation of complications at the injection site, such as infection, skin atrophy, or intense pain. The following patient-related variables were evaluated: age, gender and elbow side treated.

Minimal important differences for VAS at rest were defined for the 3 months follow-up evaluation as 1 point, as described by Challoumas et al.^11^

### Sample size

The sample was defined by convenience, defined by the total number of patients who wish to participate during the study period. The recruitment period was 3 years.

### Bias and loss of follow-up

The clinical assessment questionnaires used were applied in person, by telephone or email. Cases with missing data were treated by imputation with the last observation carried forward (when with a minimum of 3 months of follow-up) or by excluding the patient (when with no post-injection evaluation).

### Statistical analysis

We subjected continuous variables to assessment of normality, using the Kolmogorov-Smirnov test, and homogeneity, using the Levene test. The continuous data were exposed by mean and standard deviation. Categorical variables were displayed in absolute value and percentage. Comparison between sequential evaluation times was performed using Analysis Of Variance (ANOVA) with Bonferroni post-hoc test for multiple comparisons. We used p<0.05 as the significance level. For data analysis, we used the SPSS version 24.0 program (SPSS Inc®, Chicago, IL, USA).

## RESULTS

During the period evaluated, 55 patients with a confirmed diagnosis of lateral epicondylitis underwent two sequential injections of hyaluronic acid. After applying the inclusion and non-inclusion criteria, our series comprised 46 patients (52 elbows). Of these, 33 (63%) were male, 6 patients underwent to bilateral injections and 27 (52%) had only the right side treated. The mean age was 46 years ± 7.

**Figure 1:**
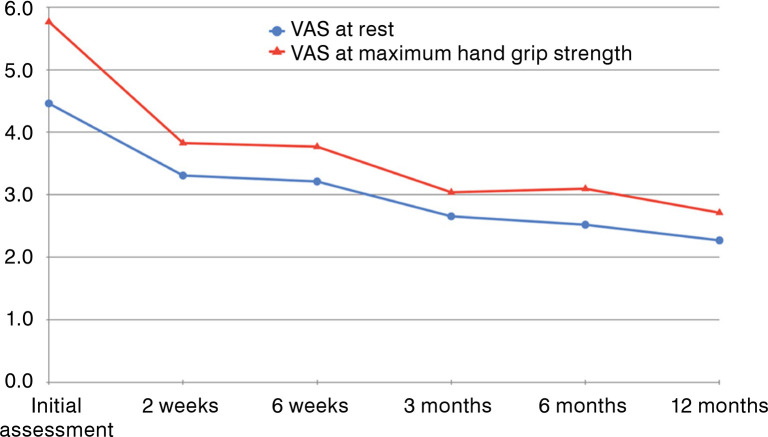
Visual analogue scale (VAS) at sequential follow-ups. VAS at rest (blue line) and VAS during maximum hand grip strength (red line).

According to the VAS at rest, the average score was 4.5 ± 2.8 in the initial assessment and 2.7 ± 2.8 at 3 months of follow-up, showing significant improvement (*p*<0.001). At 12 months of follow-up, the mean score was 2.3 ± 3.0, with statistically significant difference (*p*<0.001). In relation to the initial assessment, all periods showed statistically significant improvement (Figure 1). Regarding sequential evaluations after the initial assessment, we did not obtain a statistically significant difference. Clinical results are detailed in Table 1.

**Table 1:** Funcional evaluation at initial assessment and at sequential follow-ups.

	Mean	SD	P-value
**VAS at rest**			
Initial assessment	4.5	2.8	p<0.001*
2 weeks	3.3	2.6	
6 weeks	3.2	2.9	
3 months	2.7	2.8	
6 months	2.5	3.0	
12 months	2.3	2.9	
**VAS at maximum hand grip strength**			
Initial assessment	5.8	3.1	p<0.001**
2 weeks	3.8	2.9	
6 weeks	3.8	2.7	
3 months	3.0	3.0	
6 months	3.1	3.4	
12 months	2.7	3.3	
**SANE**			
Initial assessment	57.1	23.2	p<0.001***
2 weeks	64.5	27.3	
6 weeks	69.3	23.1	
3 months	73.2	25.8	
6 months	78.2	23.0	
12 months	77.8	23.8	

* Post-hoc analysis: difference between the follow-up times - initial assessment to all follow-ups, 2 weeks to 12 months. ** Post-hoc analysis: difference between the follow-up times - initial assessment to all follow-ups. *** Post-hoc analysis: difference between the follow-up times - initial assessment to all follow-ups, except to 2 weeks; 2 weeks to 6 and 12 months.

According to the VAS during maximum hand grip strength, the average score was 5.8 ± 3.1 in the initial assessment and 3.0 ± 3.0 at 3 months of follow-up, showing significant improvement (*p*<0.001). At 12 months of follow-up, the mean score was 2.8 ± 3.4, with statistically significant difference (*p*<0.001). In relation to the initial assessment, all periods showed statistically significant improvement.

**Figure 2:**
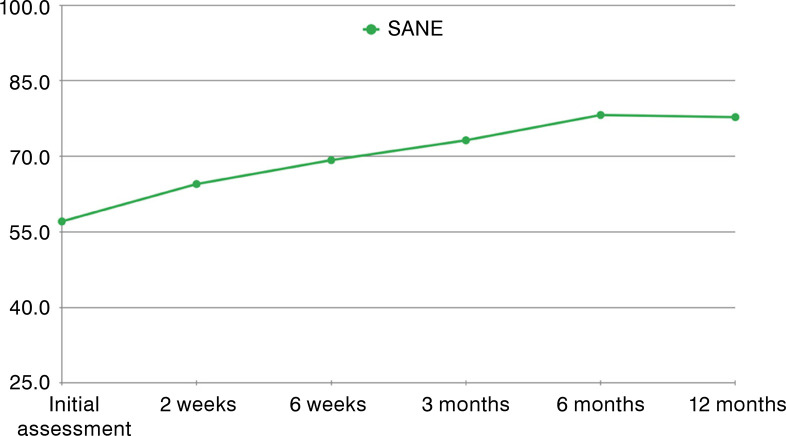
Single Assessment Numeric Evaluation (SANE) at sequential follow-ups.

SANE score also showed significant improvement after the procedure, from 57.1% ± 23.2 from the initial evaluation to 78.2% ± 23.5 (*p*<0.001) at final follow-up. In relation to the initial assessment, all periods showed statistically significant improvement, except for 2-week follow-up (Figure 2).

The results reached minimal important differences for VAS at rest in 67% of the elbows for the primary outcome. Nine elbows (17%) didn’t achieve satisfactory results after the procedure, with persistence pain (VAS at rest > 5) at final follow-up (12 months).

No patient had complications related to the procedure, such as infection or skin complications. No patient underwent further surgery.

## DISCUSSION

The use of hyaluronic acid (HA) in sport-related tendinopathies has garnered considerable attention in recent years(12). Concurrently, various HA preparations and procedural approaches are currently under scrutiny to ascertain optimal therapeutic strategies. Encouraging outcomes have also been observed in the treatment of tendinopathies, predominantly attributed to HA’s anti-inflammatory properties, augmented cellular proliferation, collagen deposition, and its lubricating effect on the tendon’s gliding surface.^13,14^ However, it is noteworthy that in the majority of studies, HA administration was not localized within the degenerated tendon itself but rather adjacent to it and/or within the articular space. This raises the possibility that alterations in synovial fluid dynamics facilitated by HA may exert a beneficial influence directly on the tendon^12^

While the literature demonstrates promising outcomes of HA therapy in the management of tendinopathies, including lateral epicondylitis, comprehensive comparative analyses evaluating the efficacy and safety profiles of various HA preparations are scarce.^15^ Moreover, the optimal dosing regimens, frequency of administration, and long-term outcomes remain to be elucidated.^15^

Over the course of our study, we observed a notable decrease in pain levels, both at rest and during maximum hand grip strength, among the majority of participants. This reduction in pain was evident not only in the short term, but also persisted up to the 12-month. Additionally, patients reported improvements in their overall satisfaction with elbow function, as demonstrated by decreases in VAS and SANE scores. The results reached minimal important differences for VAS at rest in 67% of the elbows at 3 months follow-up and as defined by Challoumas et al.^11^ However, it is important to acknowledge that not all patients experienced complete relief from their symptoms. Approximately 17% of elbows did not respond favorably to the HA injections, with some individuals still reporting significant pain even after the treatment period. Despite this, it is noteworthy that none of the patients experienced any adverse effects or complications related to the procedure, highlighting the safety profile of HA injections for lateral epicondylitis.

One study by Pellegrino et al.^16^ explored the efficacy of a combined approach involving high-intensity laser therapy (HILT) and HA injections compared to therapeutic exercise (TE). They found that the HILT + HA group exhibited significant increases in muscle strength compared to the TE group, suggesting potential benefits of HA associated with HILT in the short to medium term. On the other hand, Yalcin and Kayaalp^17^ conducted a prospective randomized controlled study comparing the efficacy of HA injections with triamcinolone injections in chronic lateral epicondylitis. They found that triamcinolone injections provided superior short-term pain relief and functional improvements compared to HA injections. Furthermore, Zinger et al.^18^ conducted a randomized controlled trial comparing HA injections with a saline control group. They reported significant success in pain relief with HA injections, which persisted up to 12 months post-injection.

In comparison to the study by Stirma et al.^19^ which also investigated the effectiveness of hyaluronic acid (HA) infiltration for lateral epicondylitis, our study had a larger sample size (12 vs 52 elbows) and a longer follow-up (3 vs 12-months). They reported a reduction in positivity rates for specific tests and improvements in the Mayo Elbow Performance Score, consistent with our observations of decreased pain levels and enhanced functional status. Moreover, both studies reported a high level of patient satisfaction with the treatment, with no complications or adverse effects observed.

Our study has some limitations. We lacked a comparative group, limiting our ability to assess HA treatment’s relative efficacy against other modalities. While lateral epicondylitis typically allows for shorter follow-ups, longer-term studies could provide a more comprehensive understanding of outcomes. Moreover, HA treatment’s higher cost may hinder accessibility for some patients. Lastly, not using a specific epicondylitis scoring system may have limited the depth of our assessment of treatment outcomes.

## CONCLUSIONS

Treatment of lateral epicondylitis with hyaluronic acid led to a statistically and clinically significant improvement for the visual analogical pain score at rest and during maximum hand grip strength and for the Single Assessment Numeric Evaluation, with no reports of complications.
